# Co-medications and dipeptidyl peptidase-4 inhibitors associated bullous pemphigoid^☆^^☆☆^

**DOI:** 10.1016/j.abd.2020.10.010

**Published:** 2021-09-30

**Authors:** Agoritsa Gravani, Panagiota Christou, Stelios Tigas, Ioannis D. Bassukas

**Affiliations:** a Department of Dermatology, University Hospital of Ioannina, Greece; b Department of Endocrinology and Diabetes Centre, University Hospital of Ioannina, Ioannina, Greece; c Department of Endocrinology and Diabetes Centre, Faculty of Medicine, School of Health Sciences, University of Ioannina, Ioannina, Greece; d Department of Dermatology, University Hospital of Ioannina, Ioannina, Greece; e Department of Skin and Venereal Diseases, Faculty of Medicine, School of Health Sciences, University of Ioannina, Ioannina, Greece

Dear Editor,

Bullous Pemphigoid (BP) is a serious cutaneous autoimmune disease with distinctly associated comorbidities such as neurological disorders, hypertension, and diabetes *mellitus*. Recently, the use of Dipeptidyl Peptidase-4 Inhibitors/gliptins (DPP4i) to control hyperglycemia was linked to the increasing prevalence of Type 2 Diabetes Mellitus (T2DM) among patients with newly diagnosed BP,^1^ demonstrating that the association of different drugs to certain comorbidities might partly account for the observed link of some comorbidities to BP. Accordingly, we asked whether medications used to treat further comorbidities might have modified the risk to develop BP in this setting. Herein, we report our findings on the association of BP with non-antidiabetic medications used at the time of BP diagnosis in a single-center cohort of elderly (≥70 years old) DPP4i-treated T2DM patients.

The BP cohort consisted of 45 T2DM patients on DPP4i with newly diagnosed BP after January 2010. BP diagnostic criteria are displayed in Table 1.^1^ All BP patients in this cohort were treated uniformly according to institutional guidelines with discontinuation of DPP4i and the combination of systemic corticosteroids in tapered doses and methotrexate used as corticosteroid-sparing agent. The controls were 98 elderly T2DM patients without BP treated with DPP4i for at least the last 30 months prior to enrollment matched at a ratio of 1:2 on gender, age (within 2 years), and year of diagnosis. Employing SPSS, categorical variables were compared with the χ^2^ test, and Mantel-Haenszel and Cox proportional hazard odds ratios (OR ± 95%Confidence Intervals, CI) were calculated between patients with and without BP at p-level <0.05. The findings are part of a retrospective study approved by the Institutional Research and Ethics Committee.

**Table 1:** Criteria for the diagnosis of Bullous Pemphigoid (BP).

All 3 criteria must be fulfilled:
(a) Relevant clinical presentation
(b) Lesional skin biopsy consistent with BP
(c) A result consistent with the diagnosis BP in at least one of the following routinely available laboratory examinations: direct immunofluorescence, indirect immunofluorescence, or ELISA

The spectrum of the employed DPP4i did not differ between patients with and without BP (p = 0.06). Core demographic and medical history data of BP patients and controls are summarized in Table 2. Drug groups were included in the analysis when at least 10/143 patients were on regular treatment using medications of each group. Evaluating these groups together, a significantly higher risk of BP was found for patients on anticoagulants, proton pump inhibitors, and selective serotonin reuptake inhibitors, whereas the BP risk was significantly lower for those T2DM patients on statins (Table 3). Furthermore, the link of statin intake with a reduced BP risk was the only association that remained significant after focusing the analysis on the four drug groups above (Cox HR = 0.165; CI = 0.038–0.723; p = 0.017). The main limitation of this study is the relatively small number of participants; however, the cohorts were reasonably homogeneous with a noticeable number of BP patients included.

**Table 2:** Core demographic characteristics and disease history data of n = 45 bullous pemphigoid patients (BP) and n = 98 controls.

Attribute	BP, n (%)	Control, n (%)
Male gender	n = 19 (42.2%)	n = 41 (41.8%)
Female gender	n = 26 (57.8%)	n = 57 (58.2%)
Age (years): Median [Range]:	80 (70–92)	76.5 (70–90)
DPP4^a^ use interval (months) – Median [Range]	16 (0.3–60)	43.5 (30–125)
Mean	19.6	53.9
**Comorbidities**		
Cardiovascular	n = 40 (88.9%)	n = 88 (89.8%)
Pulmonary	n = 6 (13.3%)	n = 7 (7.1%)
Malignancies, excluding hematologic	n = 1 (2%)	n = 0 (0%)
Psychiatric	n = 9 (20%)	n = 10 (10.2%)
Neurologic	n = 7 (15.5%)	n = 6 (6.1%)
Gastrointestinal	n = 11 (24.4%)	n = 15 (15.3%)
Metabolic (dyslipidemia, hyperuricemia)	n = 29 (64.4%)	n = 72 (73.5%)
Urogenital	n = 7 (15.6%)	n = 7 (7.1%)
Hematologic diseases, including malignancies	n = 5 (11.1%)	n = 0 (0%)
Ear Nose Throat / Eye disorders	n = 4 (8.9%)	n = 0 (0%)
Rheumatologic	n = 2 (4.4%)	n = 4 (4.1%)
Cutaneous	n = 3 (6.7%)	n = 0 (0%)
Endocrine other than diabetes mellitus	n = 4 (8.7%)	n = 10 (10.2%)

**Table 3:** Medications in patients with DPP4i associated bullous pemphigoid and in patients without BP despite DPP4i use.

Medication	Cohort, n (%)		p	OR^a^	CI^b^ for OR	
	BP (n = 45)	Controls (n = 98)			Lower	Upper
Anticoagulants	24 (53.3%)	34 (34.7%)	0.026	2.300	1.104	4.793
a1-adrenergic inhibitors	6 (13.3%)	7 (7.1%)	0.220	2.062	0.649	6.550
ACE/AT2 inhibitors^c^	36 (80%)	63 (64.3%)	0.064	2.292	0.954	5.503
Beta-blockers	18 (40%)	33 (33.7%)	0.276	1.510	0.719	3.170
Calcium channel blockers	14 (31.1%)	41 (41.8%)	0.165	0.581	0.270	1.252
Loop diuretics	11 (24.4%)	21 (21.4%)	0.444	1.393	0.596	3.170
Thiazide diuretics	20 (44.4%)	29 (29.6%)	0.065	2.009	0.958	4.214
Potassium sparing diuretics	5 (11.1%)	5 (5.1%)	0.113	3.026	0.771	11.886
Centrally acting antihypertensive drugs	4 (8%)	6 (6.1%)	0.522	1.538	0.411	5.785
Statins	21 (46.7%)	67 (68.4%)	0.010	0.376	0.179	0.790
Hypolipidemic, other	4 (8%)	8 (8.2%)	0.851	1.128	0.321	3.970
Thyroxine	3 (6.7%)	9 (9.2%)	0.643	0.725	0.186	2.823
Proton pump inhibitors	12 (26.7%)	8 (8.2%)	0.009	3.781	1.396	10.243
Benzodiazepines	6 (13.3%)	4 (4.1%)	0.051	3.730	0.995	13.982
Selective serotonin reuptake inhibitors	9 (20%)	6 (6.1%)	0.032	3.429	1.110	10.595

a Mantel-Haenszel Odds Ratio.

b CI, 95% Confidence Intervals.

c Angiotensin Converting Enzyme inhibitors & Angiotensin II receptor blockers.

Statin intake might lower the risk of developing BP in patients with T2DM treated with DPP4i by modifying certain inflammatory processes, probably via the promotion of an anti-inflammatory shift by inhibiting Th17 cells and IL-17 production.^2^ For example, in an animal model of allergic asthma, simvastatin modified the influx of inflammatory cells, including eosinophils and T_reg_, into the target tissues.^3^ Recently, Guo et al.^4^ reported a significantly increased association of the use of spironolactone with the risk of developing BP among patients taking DPP4i, even after adjustment for confounders (HR = 5.50, 95% CI = 1.25–7.51). Notably, although we did not evaluate specifically the effect of spironolactone, we could not confirm a higher BP risk for patients taking any K-sparing diuretic (Table 3). It is possible that variation of genetic factors in remote populations (like Korean vs. Greek patients), including differences in BP susceptibility and/or diverging pharmacogenomics, may explain differences in the susceptibility of the association of certain drugs to BP development.

It has been suggested that DPP4i-induced BP may become a model disease for a better understanding of basic autoimmunity principles.^5^ Targeting the role of co-medications in triggering BP in DPP4i-treated T2DM patients in prospective studies with sufficiently large patient samples might provide essential contributions to delineate missing links in the pathogenesis of this disease.

## Financial support

None declared.

## Authors' contributions

Agoritsa Gravani and Ioannis Bassukas designed the study.

Agoritsa Gravani, Panagiota Christou and Stelios Tigas collected data. All authors contributed to data analysis.

Agoritsa Gravani was responsible for the 1^st^ draft; all authors revised critically and approved the final version of the manuscript.

## Conflicts of interest

None declared.
